# Rural-urban disparity in lung function parameters of Nigerian children: effects of socio-economic, nutritional and housing factors

**DOI:** 10.11604/pamj.2017.28.230.13836

**Published:** 2017-11-15

**Authors:** Bankole Peter Kuti, Oluwatoyin Ibukun Oladimeji, Demilade Kehinde Kuti, Adewuyi Temidayo Adeniyi, Emmanuel Oluwatosin Adeniji, Yetunde Justinah Osundare

**Affiliations:** 1 Department of Paediatrics and Child Health Obafemi Awolowo University, Ile-Ife, Nigeria; 2 Department of Paediatrics Wesley Guild Hospital, Ilesa, Nigeria

**Keywords:** Lung function test, nutritional status, anthropometrics, urban, rural, school children

## Abstract

**Introduction:**

The effect of socio-demographic and nutritional factors on lung functions of African children is poorly studied. This study set out to determine the effects of these factors on lung functions of Nigerian school children.

**Methods:**

Rural and urban secondary schools students in Ilesa, Nigeria were selected by multistage sampling. The socio-demographic, nutritional status as well as lung function parameters measured using incentive Spirometry (MIR Spirolab III srl, Italy) of the children were obtained and compared among the rural and urban children.

**Results:**

A total of 250 children (128 rural and 122 urban) aged 9 to 17 years participated in the study over a 12 month period. Mean (SD) age was 12.6 (1.9) years and Male: Female 1:1.1. The urban children were heavier, taller and have larger lung volumes than their age and sex matched rural counterpart. Stunted rural males [Mean (SD) FVC 1.8 (0.3) L vs. 2.2 (0.6) L t-test = 2.360; p = 0.022], underweight females [Mean (SD) FVC 1.8 (0.4) L vs. 2.2 (0.6) L; t-test = 2.855; p = 0.006] and those exposed to unclean fuel [Mean (SD) FVC 2.1 (0.6) L vs. 2.4 (0.5) L; t-test = 2.079; p = 0.041] had significantly lower lung volumes compared to their counterparts without these conditions.

**Conclusion:**

Undernutrition, low socio-economic class and use of unclean fuels adversely affect the lung functions of Nigerian children. Improved standard of living, use of clean fuel and adequate nutrition may ensure better lung health among these children.

## Introduction

Lung function tests done using standard spirometers have been reported to be very important in care of children with chronic respiratory diseases including asthma, cystic fibrosis and interstitial lung diseases [1]. Spirometry helps in diagnosing, follow up, monitoring and objective ascertainment of response to interventions in these children [1]. Also, the usefulness of lung function assessment extends even to apparently healthy children to screen them for exercise-induced bronchospasm, as part of routine medical check-up and pre-school entry medical evaluations and pre-recruitment evaluation upon admission to certain sports and recreation activities [1,2]. The usefulness of lung function assessment also include research as part of objective end point assessment of efficacy or otherwise of certain drugs and interventions [1, 2]. Lung function parameters are however affected by various factors including anthropometric, nutritional states, ambient temperature and pressure [2,3]. Racial factors also affect the lung function parameters as Caucasians have been documented to have larger lung volumes and capacities than African Americans and Asians [3]. The place of domicile and socio-economic class children belong to, which may be a reflection of amount and duration of exposure to particulate matters and noxious gases from both indoor and outdoor air pollution, may affect the lung volumes and capacities of children [4-7]. Early onset undernutrition reported to be more prevalent in rural areas of developing countries have been reported to affect lung growth and development [8]. Conversely, overweight and obesity reported more frequently among affluent children in urban centres have also been associated with impaired lung functions [9]. Many studies to ascertain the lung function parameters of rural and urban children give variable results [4-7]. The effects of nutritional status, socio-demographic, environmental affectation of lung function parameters in apparently healthy children may vary from one location to another. Hence, there is a need to determine the lung function parameters of apparently healthy rural and urban Nigerian school children where there is paucity of data on this. This study therefore aims to determine the urban/rural disparities in the lung function parameters (FEV1, FVC and FEV1/FVC) of apparently healthy Nigerian school children and the effects of nutritional status, socio-demographic characteristics and housing conditions on lung function parameters of these children.

## Methods

**Study design**: This was a school-based analytic cross sectional study

**Study location**: This study was conducted in four secondary schools in Ilesa West Local Government Area, south West Nigeria. Ilesa (latitude 7°35'N; longitude 4°51'E) is a major semi-urban town in the state of Osun South-West Nigeria with a growing population of 620,000 people and about 40% of the population being children < 15 years [10]. The occupants of Ilesa cut across all socio-economic classes. Children from affluent upper class homes attend private schools with modern boarding facilities, while children from poorer middle and lower social class attend public schools.

**Sample selection**: The four selected schools were chosen by multistage sampling method. This was done as follows: From the two LGAs in Ilesa (West and East), Ilesa West LGA was chosen by simple random method. Ilesa west has 24 secondary schools (11 public middle schools and 13 Private schools) [11]. Two schools were selected by simple balloting from the pool of public and private schools in the LGA. Four of the 11 public schools were located in the rural parts (Mukoro) of the LGA. One school was randomly selected out of the four rural public schools. One school was also randomly selected from the 13 private schools all located in Ilesa (urban) metropolis. The selected rural public school had five arms of classes (Primary V to Junior Secondary III), while the selected urban school had six arms of classes (Junior Secondary I to Senior Secondary III). Thereafter, the children in the selected classes were proportionately recruited from each class till the required sample size was achieved for the study.

**Sample size determination**: Using 5% significance level and 90% power. Assumptions: mean difference of FEV1 among rural and urban school children = 0.36L and the standard deviation of 0.94 and 1.0L for rural and urban respectively (From the study of Sonnappa et al) [6]. Ratio of urban to rural school children to be recruited = 1:1, minimum calculated sample size was approximately 240;120 rural and 120 urban school children, would be required to be studied to detect an effect size of 0.36L FEV1; but 250 (128 rural and 122 urban) school children were studied.

**Ethical consideration**: Ethical approval for this study was obtained from the Institute of Public Health, Obafemi Awolowo University, Ile-Ife, Nigeria (HREC approval no IPH/OAU/12/254). The permission of the principals and head teachers of the participating schools as well as parental consent and the children assent were also obtained.

**Study procedure**: Using a data proforma designed for the study, information obtained from the study participants included sex, age, tribe and family size (including the number of children in the household). Overcrowding for this study was defined as having 3 or more persons sleeping in the same room as the child [12]. The socio-economic class of the study participants was determined based on ranked assessment of the parental occupation and highest educational qualification as described by Oyedeji [13]. Gainfully employed professionals were sub classified as upper class, artisans with secondary education were sub classified as middle class, while peasant farmers, petty traders with primary or no formal education were sub classified into low social class [13]. The types of fuel used for household cooking, heating and lighting were also recorded. Kerosene, firewood, crop residues and other biomass fuels were classified as unclean fuels, while electric and cooking gas were classified as clean fuels [14, 15]. The presence of household pets, domestic animals and poultry was recorded.

**Anthropometric measurements**: The weights of the study participants were measured using a weighing scale (Leaidal Medical Ltd, United Kingdom (UK)) which measures weight to the nearest 0.5kg. The height of the children was measured to the nearest 0.5cm using a RGZ-160 stadiometer (Leaidal Medical Ltd, United Kingdom (UK)). Each study participant stood erect without shoes on the stadiometer. The children were required to put their feet together with their heels, buttocks and occiput touching the wall while they look straight ahead to ensure accurate measurement of their height. The body mass index (BMI) in kg/m^2^ was calculated from the formula: (weight in kg)/(height in m)^2^. The World Health Organisation (WHO) growth reference chart was used to determine the nutritional status of the study participants by comparing their anthropometrics with the WHO standard [16]. For this study, stunting and severe stunting were defined as height for age less than 15^th^ and 3^rd^ centiles respectively. Underweight was defined as BMI less than 15^th^ centile and overweight as BMI > 85^th^centile on WHO growth reference chart [16]. All the study participants had lung function tests using Spirolab III spirometer (MIR Spirolab III Medical International Research srl Italy) following ATS/ERS recommendation [2,3]. The procedure was explained and demonstrated to all the children. The children were comfortably seated on a chair, they were instructed to inhale to maximum capacity (total lung capacity), nose clips were applied, they were then instructed to exhale as fast and as long as possible (to residual volume). This was done through a mouthpiece into the spirometer to get the required parameters [3]. The lung function parameters of interest for this study were Forced Expiratory Volume in one second (FEV1), Forced Vital Capacity (FVC) and the Tiffeneau index FEV1/FVC. The predicted values used for this study were based on the data of Knudson et al [17]. Each study participant had a minimum of three readings and a maximum of eight readings as recommended by the ATS/ERS [2, 3]. The best reading out of those that met the acceptability and or usability criteria was used for the interpretations of the lung function parameters [2, 3]. A minimum of three flow-volume loop results within 150 ml (100 ml if FVC < 1.0 L) of highest and next highest FVC were recorded and the flow-volume loop with the highest FEV1 was analyzed. Children previously diagnosed with asthma, those with any form of respiratory diseases, those with chest deformity, dwarfism and those who couldn't perform an acceptable or usable lung function tests were excluded.

**Quality control**: The lung function test was done using incentive Spirometry to instruct as well as encourage the children to do acceptable or at least usable tests following ATS/ERS guidelines [2, 3]. Also the tests were carried out between 10:00am to 12:00 noon each day to limit the effects of ambient temperature changes on the readings and to ensure the baseline test was done before the children were involved in any physical activity. Calibration checks were carried daily to ensure high quality readings by the spirometer during the study period.

**Data analysis**: This was done using Statistical Program for Social Sciences (SPSS) software Version 17.0 (SPSS Inc, Chicago 2008). Continuous variables such as ages, weight, height FEV1 and FVC were tested for normality and summarized using mean and standard deviations (SD) for all normally distributed variables. Proportions and percentages were determined for categorical variables such as sex, age categories and nutritional status. Differences between the means (SD) lung function parameters were analyzed using Student's t-test, whereas categorical variables were analyzed using Pearson's Chi-square test and Fisher's exact test, as appropriate. Pearson's correlation tests were performed to determine the relationship between anthropometric measurements of the children and their lung function parameters. Level of significance at 95% confidence interval (CI) was taken at P < 0.05.

## Results

**Socio-demographic and general information of the study participants**: Details about the socio-demographic characteristics, housing condition and general information of the rural and urban school children recruited for the study are highlighted in Table 1. A total of 285 school children participated in the study, 35 (12.3%) could not perform an acceptable or usable spirometer tests hence were excluded. Two hundred and fifty (128 rural and 122 urban) children form the basis of further data analysis.

**Table 1:** Socio-demographic and general information of the rural and urban school children

Variables	Rural childrenn = 128 (%)	Urban childrenn =122 (%)	Total 250	p-value
Sex				
Male	55 (43.0)	68 (55.7)	123	0.549
Female	73 (57.0)	54 (44.3)	127	
Age (in years)				
9 -11	29 (22.6)	24 (19.7)	53	0.564
12-14	81 (63.3)	78 (63.9)	159	0.915
15-17	18 (14.1)	20 (16.4)	38	0.608
Mean (SD) age	12.7 (1.8)	12.4 (2.0)		0.153^#^
Tribe				
Yoruba	118 (92.2)	111 (91.0)	229	0.752
Ibo	7 (5.5)	8 (6.6)	15	0.867
Others^!^	3 (2.3)	3 (2.5)	6	0.762
Number of children in household				
≤3	21 (16.4)	50 (41.0)	71	<0.001
>3	107 (83.6)	72 (59.0)	179	
Mean (SD)	4.8 (1.6)	3.9 (1.5)		<0.001#
Socio-economic class				
Upper	0.0 (0.0)	72 (59.0)	73	<0.001
Middle	62 (48.4)	42 (34.4)	103	<0.001
Lower	66 (51.6)	8 (6.6)	74	<0.001
Overcrowding				
Yes	73 (57.0)	35 (28.7)	108	<0.001
No	55 (43.0)	87 (71.3)	142	
Fuel for household cooking*				
Clean fuel	16 (12.5)	69 (56.6)	82	<0.001
Unclean fuel	112 (87.6)	53 (34.7)	165	
Type of unclean fuel				
Kerosene	91 (71.1)	46 (37.7)	137	<0.001
Others	37 (20.9)	76 (62.3)	113	
Firewood	19 (14.8)	6 (4.9)	25	0.009
Others	109 (85.2)	116 (95.1)	225	
Smokers in household				
Yes	4 (3.1)	4 (3.3)	8	0.945
No	124 (96.9)	198 (96.7)	242	
Keep household pets				
Yes	52 (40.6)	49 (40.2)	101	0.983
No	76 (59.4)	73 (59.8)	149	

* Clean fuel – gas and electric; unclean fuel – kerosene, coals, firewood and other biomass fuel.

# Independent t-test applied.

! Others are Urhobos, Ebiras

**Age and sex**: The mean (SD) age of the school children was 12.5 (1.9) years which ranged from 9 to 17 years. No significant difference in the ages of the children in rural (12.7 (1.8) years) and urban schools (12.4 (2.0) years). Likewise, no difference was observed in the sex distribution of the children. The male to female ratio of the school children was 0.9:1.

**Tribe**: Majority (91.6%) of the children were Yoruba which is the predominant tribe in the study location. Other tribes represented are highlighted in Table 1.

**Rural-urban difference as related to socio-economic class, housing and home environment**: Significantly more proportions of children from rural schools were from low and middle social class compared to those from urban schools. (Table 1) Number of children in household: the mean (SD) number of children in each household of the study participants was 4.4 (1.7) children which ranged from one to 13 children per household. Significantly, more rural than urban households had more children (Table 1) Significantly more proportions of rural children lived in overcrowded homes; use unclean fuels and biomass fuels compared to the urban children. Other housing conditions as related to the rural and urban children are highlighted in Table 1.

**Nutritional status, anthropometric indices and lung function parameters of the study participants**: The rural-urban disparities in anthropometric indices, nutritional factors and lung function parameters in the male and female study participants are highlighted in Table 2 and Table 3

**Table 2:** Anthropometric indices, nutritional status and lung function parameters of the study participants

Variables	Rural children n=128 (%)	Urban childrenn =122 (%)	t-test	p-value
Anthropometric indices				
Weight mean (SD) kg	36.2 (8.7)	40.1 (11.0)	3.133	0.002
Height mean (SD) m	1.46 (0.11)	1.48 (0.18)	1.238	0.217
Body Mass Index (Kg/m^2^)	16.65 (2.3)	17.43 (2.5)	2.715	0.007
Lung function parameters				
FEV_1_ (s) Mean (SD)	1.79 (0.46)	1.99 (0.54)	3.150	0.002
FEV_1_ % Mean (SD)	78.5 (15.0)	86.5 (17.5)	3.890	<0.001
FVC (s) Mean (SD)	2.11 (0.57)	2.45 (0.77)	3.845	<0.001
FVC% Mean (SD)	80.5 (14.5)	88.5 (12.5)	4.480	<0.001
FEV_1_/FVC (%) Mean (SD)	85.20 (9.78)	82.98 (11.20)	1.665	0.097
Nutritional status			*x^2^	
Overweight/Obese	8 (6.2)	12 (10.9)	1.091	0.296
Not overweight/obese	120 (93.8)	110 (89.1)		
Underweight	31 (24.2)	13 (10.7)	7.923	0.005
Not underweight	97 (75.8)	109 (89.3)		
Stunting	21 (16.4)	11 (9.0)	3.056	0.080
Not stunted	107 (83.6)	111 (91.0)		

* Chi squared test applied

**Table 3:** Rural-urban difference in the anthropometrics and lung function parameters of the Male and female study participants

Variables	Rural male children Mean (SD)	Urban male childrenMean (SD)	Mean difference (95% CI)	t-test	p-value
Weight (Kg)	35.2 (7.6)	39.0 (11.0)	-3.8 (-7.3 to -0.3)	2.151	0.033
Height (m)	1.4 (1.1)	1.5 (1.3)	-0.1 (-0.02 to 0.1)	2.114	0.037
BMI (kg/m2)	16.5 (2.0)	17.0 (2.3)	-0.5 (-1.3 to 0.3)	1.187	0.237
FEV1 (l)	1.8 (0.5)	2.0 (0.5)	-0.2 (-0.4 to -0.04)	2.483	0.014
FVC (l)	2.1 (0.6)	2.5 (0.8)	-0.4 (-0.7 to -0.1)	3.123	0.002
FEV1/FVC	85.7 (9.9)	82.2 (10.9)	3.5 (1.3 to 7.2)	1.842	0.068
Variables	Rural female children Mean (SD)	Urban female children Mean (SD)	Mean difference (95% CI)	t-test	p-value
Weight (Kg)	36.9 (9.3)	41.5 (10.9)	-4.6 (-8.2 to -1.0)	2.559	0.012
Height (m)	1.5 (1.0)	1.5 (0.1)	-0.03 (-0.07 to -0.01)	1.556	0.122
BMI (kg/m2)	16.7 (2.5)	18.1 (2.6)	-1.4 (-2.3 to -0.5)	2.977	0.004
FEV1 (l)	1.8 (0.5)	1.9 (0.6)	-0.1 (-0.3 to -0.01)	1.763	0.080
FVC (l)	2.1 (0.6)	2.3 (0.70)	-0.2 (-.04 to -0.01)	1.98	0.050
FEV1/FVC	84.8 (9.7)	84.0 (11.50)	0.8 (0.1 to 4.0)	0.423	0.673

**Weight and height of the study participants**: The mean (SD) weight of the 250 school children was 38.0 (10.0) kg which ranged from 19.0kg to 68.0kg. Their heights ranged from 1.2m to 1.8 m with a mean (SD) height of 1.5 (0.1)m. The urban children were heavier and taller than their rural counterparts. Likewise their body mass index was significantly higher than that of their rural counterpart. (Table 2)

**Nutritional status of the study participants**: Significantly, more rural children were underweight (p = 0.002) compared to their urban counterpart. Though more proportions of the rural children were stunted compared to the urban children, the difference was not significant.

**Lung function parameters of the study participants**: The mean (SD) FEV1 of the 250 school children was 1.9 (0.5) litres (L) which ranged from 0.8 to 3.5L. The mean (SD) FEV1% was 82.5 (17.5)%. The mean (SD) FVC was 2.3 (0.7) L which ranged from 0.8 to 4.8 L. The mean (SD) FVC% was 86.5 (11.5)%. The mean (SD) FEV1 and FVC of the male urban children were significantly higher than that of their rural counterpart. However for the females, only FVC was significantly higher in urban females than the rural females (Table 3).

**Correlations of anthropometric indices with lung function parameters**: In addition to the age, anthropometric parameters like weight, height and body mass index were positively correlated with the FEV1 and FVC of the children (p < 0.05) Table 4. Lung function parameters (FEV1 and FVC) correlated better with age and anthropometric indices among the urban male children than the rural male children; while among the female children lung function parameters correlated better with the anthropometric indices of the rural than the urban female children (Table 4).

**Table 4:** Correlation between anthropometric indices and lung function parameters of the male and female children

	Male Rural children				Male Urban Children				
Variables	Correlation coefficient with FEV_1_	p-value	Correlation coefficient with FVC	p-value	Correlation coefficient with FEV_1_	p-value		Correlation coefficient with FVC	p-value
Age (years)	0.663	<0.001	0.653	<0.001	0.429	0.001		0.455	0.001
Weight (kg)	0.837	<0.001	0.710	<0.001	0.684	<0.001		0.644	<0.001
Height (m)	0.861	<0.001	0.748	<0.001	0.695	<0.001		0.660	<0.001
BMI (kg/m^2^)	0.590	<0.001	0.490	<0.001	0.338	0.013		0.735	0.013
Tiffeneau index	-0.137	0.263	-0.596	<0.001	0.128	<0.357		-0.336	0.013
	Female Rural children				Female Urban Children				
Variables	Correlation coefficient with FEV_1_	p-value	Correlation coefficient with FVC	p-value	Correlation coefficient with FEV_1_	p-value		Correlation coefficient with FVC	p-value
Age (years)	0.408	<0.001	0.400	<0.001	0.416	0.002		0.379	0.005
Weight (kg)	0.642	<0.001	0.630	<0.001	0.557	<0.001		0.479	<0.001
Height (m)	0.651	<0.001	0.619	<0.001	0.562	<0.001		0.565	<0.001
BMI (kg/m^2^)	0.522	<0.001	0.529	<0.001	0.451	0.001		0.302	0.030
Tiffeneau index	0.133	0.260	-0.287	0.013	0.139	0.322		-0.331	0.016

Association between socio-demographic variables, housing condition, nutritional status and lung function parameters of the rural and urban children: Details of the association of these variables and lung function parameters of the male and female rural and urban children are highlighted in Table 5 and Table 6. Significantly urban male children from low socio-economic class had lower FEV1 and FVC than those from middle and higher class. (>Mean (SD) FEV1 1.6 (0.4) vs. 2.1 (0.5) L; t-test = 2.384; p = 0.022) and (Mean (SD) FVC 1.9 (0.5) L vs. 2.6 (0.8) L; t-test = 2.057; p = 0.022). Likewise, stunting was significantly related to lower lung volume and flow among the rural male children [Mean (SD) FEV1 1.6 (0.2) L vs. 1.9 (0.5) L; t-test = 2.351; p = 0.023) and (Mean (SD) FVC 1.8 (0.3) L vs. 2.2 (0.6) L; t-test = 2.360; p = 0.022) Among the female rural children, use of unclean fuel (Mean (SD) FVC 2.1 (0.6) L vs. 2.4 (0.5) L; t-test = 2.079; p = 0.041) and underweight (Mean (SD) FEV1 1.5 (0.4) L vs. 1.9 (0.6) L t-test = 3.070; p = 0.003) and (Mean (SD) FVC 1.8 (0.4) L vs. 2.2 (0.6) L; t-test = 2.855; p = 0.006) were significantly related to low lung volumes (Table 6).

**Table 5:** Socio-demographic variables and nutritional status as related to the lung function parameters of the male rural and urban study participants

	Male rural children				Male urban children			
Variables	FEV1(L)Mean (SD)	p-values	FVC (L) Mean (SD)	p -values	FEV1(L)Mean (SD)	p-values	FVC (L) Mean (SD)	p -values
Social class								
Upper & middle	1.9 (0.5)	0.151	2.3 (0.7)	0.059	2.1 (0.5)	0.044	2.6 (0.8)	0.022
Low	1.7 (0.4)		2.0 (0.5)		1.6 (0.4)		1.9 (0.5)	
Housing								
Overcrowding	1.8 (0.5)	0.692	2.1 (0.6)	0.697	1.9 (0.4)	0.330	2.5 (0.7)	0.649
No overcrowding	1.8 (0.5)		2.2 (0.6)		2.1 (0.8)		2.6 (0.8)	
Household pets	1.8 (0.5)	0.628	2.1 (0.4)	0.971	2.2 (0.5)	0.072	2.8 (0.8)	0.091
No pets	1.8 (0.4)		2.1 (0.7)		2.1 (0.5)		2.4 (0.8)	
Children in household								
>3	1.8 (0.4)	0.537	1.8 (0.7)	0.913	1.9 (0.5)	0.475	2.5 (0.8)	0.973
≤3	1.9 (0.6)		2.1 (0.6)		2.1 (0.6)		2.5 (0.9)	0.475
Household fuel								
Clean fuel	1.8 (0.5)	0.027	2.2 (0.6)	0.113	2.1 (0.5)	0.241	2.7 (0.7)	0.296
Unclean fuel	1.4 (0.4)		1.7 (0.6)		1.9 (0.6)		2.4 (0.9)	
Smokers								
Yes	1.8 (0.9)	0.875	1.8 (0.7)	0.272	2.1 (0.9)	0.702	2.9 (0.9)	0.360
No	1.8 (0.4)		2.1 (0.6)		2.0 (0.5)		2.5 (0.8)	
Nutritional status								
Stunting	1.6 (0.2)	0.023	1.8 (0.3)	0.022	1.8 (0.5)	0.139	2.1 (0.6)	0.124
No stunting	1.9 (0.5)		2.2 (0.6)		2.1 (0.5)		2.6 (0.8)	
Underweight	1.6 (0.3)	0.071	1.9 (0.5)	0.125	1.8 (0.3)	0.218	2.3 (0.6)	0.256
No underweight	1.9 (0.5)		2.2 (0.6)		2.1 (0.6)		2.6 (0.8)	
Overweight	2.1 (0.8)	0.329	2.3 (0.8)	0.668	2.2 (0.5)	0.558	3.0 (1.1)	0.186
No overweight	1.8 (0.50		2.1 (0.6)		2.0 (0.5)		2.5 (0.8)	

Bold figures signifies statistical significance

**Table 6:** Socio-demographic variables and nutritional status as related to the lung function parameters of the female rural and urban study participants

	Female rural children				Female urban children			
Variables	FEV1(L)Mean (SD)	p-values	FVC (L) Mean (SD)	p -values	FEV1(L)Mean (SD)	p-values	FVC (L) Mean (SD)	p -values
Social class								
Upper & Middle	1.8 (0.5)	0.612	2.1 (0.6 )	0.790	1.4 (0.5)	0.337	1.5 (0.6)	0.223
Low	1.8 (0.4)		2.1 (0.5)		2.0 (0.6)		2.4 (0.7)	
Housing								
Overcrowding	1.8 (0.4)	0.834	2.1 (0.5)	0.077	1.9 (0.8)	0.784	2.3 (0.8)	0.590
No overcrowding	1.8 (0.5)		2.1 (0.7)		2.0 (0.5)		2.4 (0.7)	
Household pets	1.8 (0.4)	0.758	2.1 (0.5)	0.441	1.9 (0.7)	0.792	2.4 (0.8)	0.877
No pets	1.8 (0.5)		2.2 (0.6)		2.0 (0.5)		2.3 (0.6)	
Children in household								
>3	1.8 (0.5)	0.542	2.1 (0.4)	0.926	1.8 (0.6)	0.272	2.2 (0.7)	0.349
≤3	1.7 (0.4)		2.1 (0.6)		2.0 (0.5)		2.4 (0.7)	
Household fuel								
Clean fuel	2.0 (0.4)	0.100	2.4 (0.5)	0.041	2.0 (0.6)	0.791	2.4 (0.8)	0.599
Unclean fuel	1.7 (0.5)		2.1 (0.6)		1.9 (0.5)		2.3 (0.6)	
Smoker in household								
Yes	1.8 (0.5)	0.179	2.1 (0.6)	0.676	1.9 (0.8)	1.000	2.1 (0.8)	0.730
No	2.4 (0.3)		2.4 (0.6)		1.9 (0.6)		2.3 (0.7)	
Nutritional status								
Stunting	1.6 (0.4)	0.202	1.8 (0.6)	0.082	1.9 (0.8)	0.789	2.1 (0.7)	0.500
No stunting	1.8 (0.5)		2.2 (0.5)		1.9 (0.6)		2.4 (0.7)	
Underweight	1.5 (0.4)	0.003	1.8 (0.4)	0.006	1.7 (0.3)	0.534	2.3 (0.7)	0.858
No underweight	1.9 (0.6)		2.2 (0.6)		2.0 (0.6)		2.4 (0.9)	
Overweight	1.7 (0.4)	0.580	2.0 (0.7)	0.615	2.0 (0.7)	0.565	2.4 (0.8)	0.816
No overweight	1.8 (0.5)		2.1 (0.6)		1.9 (0.5)		2.3 (0.7)	

Bold figures signifies statistical significance

## Discussion

This study had demonstrated significant larger lung volumes and capacities (FEV1 and FVC) among urban than age and sex-matched rural Nigerian school children. Also highlighted from this study is that urban male children from low socio-economic class, undernourished rural children and those exposed to noxious particulate and gaseous substances from the use of unclean fuels have significantly lower lung function parameters than their age and sex matched counterparts. Undernutrition was more prevalent among the rural school children than their urban counterpart. Among the rural children one in five was underweight compared to one in 10 urban children. Also 16.4% and 9.0% of rural and urban children were stunted respectively. This higher prevalence of undernutrition among rural compared to urban children was also reported by Senbajo et al [18] and Ijarotimi et al [19] from southwest Nigeria, Tadesse et al [20] from Ethiopia and from other parts of sub-Saharan Africa and Asia as reported by UNICEF in her global report on nutrition 2016 [21]. Children from rural areas are socially disadvantaged with limited access to basic necessities of life including food and quality health care [21]. These predispose them to recurrent infections and infestations with undernutrition being one of the numerous consequences [22]. In addition to poor access to food and qualitative health care, children from rural areas are often from low socio-economic class live in poorly ventilated and crowded homes [23, 24] and are exposed to indoor air pollution through the use of unclean fuel and biomass for cooking, lightening and heating [14, 15]. In the present study, higher proportion of children from rural areas are exposed to indoor air pollution, lived in overcrowded homes and use unclean fuels for household cooking. These finding were similarly reported by Fotso [23] and was highlighted by the WHO in her reports on rural-urban as well as intra-urban health inequalities [24]. This call for policies, programmes and political will to improve the standard of living of the rural and urban slumps dwellers, improve quality of life in these places to ensure good nutritional status and better lung health. The higher lung volumes among urban children than their age and sex matched rural counterparts observed in this study is similar to reports by other workers. [4-6] This may be related to the fact that urban children are often taller and well-nourished than their rural counterpart as shown in this study and similar ones [4-6]. As lung volumes are related to height and weight, taller children definitely will have higher lung volumes and capacities [1-3]. Children from low socioeconomic class among the urban male children were found to have significantly lower lung volumes and capacities than those from middle and high social class. This observation was reported by other workers in developed [4] and developing countries [5-7].

The explanations for this observation may be due to multiple inter-related factors including perinatal, environmental and nutritional factors as well as accessibility to qualitative health care. Hegewald and Crapo [25] in a review of published works on the effects of socio-economic class on the lung function of children and adults proposed a number of associated factors. These include exposure to noxious substances in-utero from maternal inhalation of particulate matters and gases from unclean fuel cooking fuels and other pollutants more commonly seen in women from low socio-economic class [25]. Adverse perinatal events like perinatal asphyxia which are also more common among babies from low socio-economic class may adversely affect lung development of the foetus ultimately resulting in reduced lung function parameters [25]. Furthermore recurrent childhood respiratory tract infections and infestations commonly observed among children from low social class may also contribute to their having lower lung functions [25]. Likewise suboptimal housing environment like overcrowded homes commonly associated with low socio-economic classes have been associated with significantly low lung volumes and capacities [26]. These inter-related factors associated with low socioeconomic class have been found to significantly impair lung health. This implies that improving the standard of living of the populace, provision of safe cooking stove and reducing household air pollution will ultimately improve lung health particularly among the less privileged in urban slums and rural areas [27]. Worthy of note from this study is that female rural children exposed to the use of unclean fuels for household cooking had significantly lower lung function than those who used clean fuels. However this observation was not the same among male rural children. This may be related to the gender based role of the females who are often required to cook or assist in the cooking of the family daily meals using these unclean fuels thus more likely to be exposed to particulate matters and noxious gases from the burning of these fuels. This gender based exposure to particulate matters and noxious gases from the burning of unclean fuels had also been reported by Rinne et al [28] from Ecuador. Gauderman et al [29] estimated that there is a cumulative reduction of 3.4% in FEV and 5.0% in MMEF over the 4-yr study period among Californian children exposed to ambient air pollution compared to those not exposed to the pollution. Unfortunately, a large proportion of households in developing countries including Nigeria still use unclean sources of fuels for cooking. In the present study over 87 percent of the rural children and about one-thirds of the urban children use unclean fuels for household cooking. This substantiates the report by the WHO that up to 90 percent of rural dwellers in low and medium income countries (LMICs) depend on biomass and other unclean fuels for cooking lighting and heating [27]. These predispose them to all different forms of illness and diseases including respiratory diseases with ultimate impaired lung functions [14-15, 27]. Making affordable and accessible sources of clean fuels accessible to household particularly in rural areas of LMICs will ensure better lung health and higher quality of life.

Exposure to tobacco smoke either directly or passively has been related to impaired lung functions in children and adolescents [30, 31]. However, no significant association was found in this study between exposure to passive smoking among the children and their lung function parameters. This may be because only 3.2 percent of the children were exposed to passive cigarrete smoking in their household. This low prevalence of household smokers in Nigeria had also been reported by other workers [32, 33]. Undernutrition has been reported to impair lung function in children [8]. In the present study among the rural children stunted males and underweight females significantly had lower lung volumes and capacities than their well-nourished counterparts who were also from rural areas. These observations were similarly reported among apparently healthy undernourished children [34, 35] and those with cystic fibrosis [36]. Association between undernutrition and impaired lung health may be a cause-effect one. While children with lung diseases may have low lung volumes and capacities ultimately leading to poor growth and undernutrition, [37] children with primary undernutrition may have relative weakness and wasting of their respiratory muscles resulting in reduced ventilatory strength and capacities [38]. This emphasise the need to combat childhood undernutrition through targeted nutritional supplementation programmes like school meal programmes to ensure improved childhood lung health [39]. Worthy of note however is the fact that no association was observed among the overweight and obese children and their lung function parameters compared to the well-nourished children in this study. This contrasts with findings from a review by Tenório et al [9]. This may be related to the very small proportion of overweight/obese children (10.9 and 6.2 percent for urban and rural respectively) in the study population. Childhood obesity being an emerging form of malnutrition in developing countries, calls for more studies to ascertain the effects of overweight and obesity on lung functions of children. This study used standard spirometer ((MIR Spirolab III srl, Italy) with visual incentive spirometry applications to encourage the children to do an acceptable and or usable spirometry tracings according to ATS/ERS recommendation. We also related the lung function parameters of the rural and urban children to a number of socio-demographic, housing and nutritional factors. These constitute strengths of the study. We however appreciate the limitations that the quantification of the extent of exposure to household air pollutants the children were exposed to, through specific measurement of particulate matters (PM2.5) and noxious gases was also not done in this study due to none availability of facilities to do so. Nonetheless, this study may serve as a basis for further research on the effects of socio-economic, household air pollution and undernutrition of lung functions parameters of apparently healthy rural and urban Nigerian school children.

## Conclusion

We report a significantly lower lung function parameters (FEV1 and FVC) among rural Nigerian children compared to their age and sex matched urban counterparts. Also, urban children from low socio-economic class, undernourished rural children and those exposed to household air pollution from the use of unclean cooking fuels have significantly lower lung function parameters. We recommend provision of cleaner fuels and reduction of childhood undernutrition and poverty particularly among rural dwellers to improved lung health.

### What is known about this topic

- Lung function test using standard spirometer is an important tool for diagnosis and monitoring of children with various lung diseases;
- Disparities exist in the lung function parameters of urban and rural children;
- Lung function parameters are affected by nutritional and environmental factors.

### What this study adds

- Rural children have significantly lower lung function parameters compared to their age and sex-matched urban counterparts;
- Female rural children exposed to unclean cooking fuels have lower lung volumes and capacities than those whose household used clean fuels;
- Stunted males and underweight females have lower lung volumes and flows compared to their well-nourished counterparts. Therefore combating undernutrition and indoor air pollution may improve lung health in children.

## Competing interests

The authors declare no competing interests.
